# Citrullinated vimentin as an important antigen in immune complexes from synovial fluid of rheumatoid arthritis patients with antibodies against citrullinated proteins

**DOI:** 10.1186/ar3070

**Published:** 2010-07-07

**Authors:** Katleen Van Steendam, Kelly Tilleman, Marlies De Ceuleneer, Filip De Keyser, Dirk Elewaut, Dieter Deforce

**Affiliations:** 1 Laboratory for Pharmaceutical Biotechnology, Ghent University, Harelbekestraat 72, B-9000 Ghent, Belgium; 2 Department of Rheumatology, Ghent University Hospital, De Pintelaan 185, B-9000 Ghent, Belgium

## Abstract

**Introduction:**

Rheumatoid arthritis (RA) is an inflammatory disease, which results in destruction of the joint. The presence of immune complexes (IC) in serum and synovial fluid of RA patients might contribute to this articular damage through different mechanisms, such as complement activation. Therefore, identification of the antigens from these IC is important to gain more insight into the pathogenesis of RA. Since RA patients have antibodies against citrullinated proteins (ACPA) in their serum and synovial fluid (SF) and since elevated levels of citrullinated proteins are detected in the joints of RA patients, citrullinated antigens are possibly present in IC from RA patients.

**Methods:**

IC from serum of healthy persons, serum of RA patients and IC from synovial fluid of RA patients and Spondyloarthropathy (SpA) patients were isolated by immunoprecipitation. Identification of the antigens was performed by SDS-PAGE, mass spectrometry and immunodetection. The presence of citrullinated proteins was evaluated by anti-modified citrulline (AMC) staining.

**Results:**

Circulating IC in the serum of RA patients and healthy controls contain fibrinogenβ and fibronectin, both in a non-citrullinated form. Additionally, in IC isolated from RA SF, fibrinogenγ and vimentin were identified as well. More importantly, vimentin and a minor portion of fibrinogenβ were found to be citrullinated in the isolated complexes. Moreover these citrullinated antigens were only found in ACPA+ patients. No citrullinated antigens were found in IC from SF of SpA patients.

**Conclusions:**

Citrullinated fibrinogenβ and citrullinated vimentin were found in IC from SF of ACPA+ RA patients, while no citrullinated antigens were found in IC from SF of ACPA- RA patients or SpA patients or in IC from serum of RA patients or healthy volunteers. The identification of citrullinated vimentin as a prominent citrullinated antigen in IC from SF of ACPA+ RA patients strengthens the hypothesis that citrullinated vimentin plays an important role in the pathogenesis of RA.

## Introduction

Rheumatoid arthritis (RA) is a progressive autoimmune disease characterized by chronic inflammation of the peripheral joints. It is a complex multifactorial pathology, in which genetic and environmental factors, like smoking, can play an important role in the onset of disease and the progression of the joint damage [1,2]. The presence of immune complexes (IC) in serum and synovial fluid (SF) of RA patients is likely to contribute to the pathogenesis of the disease and to articular damage, since they are responsible for the activation of complement, the stimulation of phagocytes through their Fc receptor and the release of chemotactic factors, cytokines, metalloproteinases and reactive oxygen intermediates [3-6]. The formation of IC as such is not specifically related to autoimmune pathologies as it is a natural process, completing an immune response in the body. The antigen-antibody complexes are usually effectively removed by phagocytosis. However, it is known that an impaired clearance of these complexes can elicit or sustain an inflammatory response [7,8].

The pathological nature of IC has been suggested by several groups based on *in vitro *studies. The effect of the SF IC from juvenile RA patients on healthy PBMCs was studied by Jarvis *et al*. They found that especially the high molecular weight IC, separated by size exclusion chromatography from the other immunoglobulins and low molecular weight IC, were responsible for inducing a spectrum of pro-inflammatory cytokines, such as TNFα, IL-1β, IL6, IL8 and granulocyte-macrophage colony-stimulating factor (GM-CSF) [9]. A comparison between IC from SF of RA patients, serum of RA patients and serum of healthy persons was made by Schuerwegh *et al*. They demonstrated that IC isolated from RA serum and RA SF, in contrast to IC from healthy persons, had an effect on chondrocyte growth, NO production and apoptosis, thereby contributing directly to cartilage destruction in RA [10]. Mathsson *et al*. showed that polyethylene glycol (PEG) precipitated IC from RA SF induced the production of the pro-inflammatory cytokine TNFα in peripheral blood mononuclear cell (PBMC) cultures from healthy donors. When IC from RA serum or healthy serum were used, no elevated levels in TNFα could be seen [11]. These reports show the relevance of IC in the joint destruction and the pathogenesis of RA.

The best known IC in RA is the rheumatoid factor (RF) bound to its antigen, the Fc domain of IgG. The RF, which is mainly IgM [12], is used in diagnostic tests for RA and has a sensitivity of 78.6% and a specificity of 80.8% [13]. The RF factor is also found in other diseases such as systemic sclerosis (20 to 30%) [14] and occasionally in healthy persons (1.3 to 4%) [5]. Besides the RF, immunoglobulins and complement factors, other components can also be present in IC from serum of RA patients. Indeed, recently, it has been shown that fibrinogen and citrulline-containing fibrinogen are present in the IC of RA patients [15]. Because of the pathogenic nature of IC in RA, it is important to identify the antigens in these complexes. After identification of these antigens, a better understanding of the immunological process in the affected joints can be achieved.

Since anti-citrullinated protein/peptide antibodies (ACPA) are very specific for RA (specificity of 98%, sensitivity 68%) [16] and high amounts of citrullinated proteins, like fibrinogen, have been detected in the joint of RA patients, it is likely that some antigens in IC of RA patients are citrullinated.

The isolation of IC and subsequent identification of the antigens is therefore of great importance in the understanding of RA. The isolation of IC from biological matrices has been tackled by many different techniques such as PEG precipitation [10,11,17], C1q ELISA [15] and immunoprecipitation [18]. PEG precipitation is broadly used for the isolation of IC but the IC-fraction also contains a considerable amount of non immune complex (IC)-related proteins, such as albumine, haptoglobin and α1-antitrypsin [17]. C1q ELISA will isolate IC that are bound to the C1q component of the complement and this method is gaining interest because of the high throughput possibilities. However, to capture IC by C1q ELISA, C1q must be present and accessible in the IC. IC from serum and SF can be isolated with a high purity by means of immunoprecipitation with proteinG, but it has the disadvantage of isolating the free immunoglobulins as well. For the identification of the antigens in IC, a sensitive method like mass spectrometry and immunodetection is necessary because of their low abundance.

In this report a broad range proteome approach, by means of mass spectrometry, is used in order to find new antigens in IC. Because of the low abundance of the antigens and the excess of immunoglobulins, it is possible that not all antigens will be detected by this approach, especially antigens that have a molecular weight that correspond to those of immunoglobulins. Therefore, a second, very sensitive method such as immunodetection on 2D-PAGE, was chosen to confirm the results of the broad range proteome approach and to investigate whether known antigens in RA (e.g. fibrinogenβ (Fibβ), fibrinogenγ (Fibγ), fibronectin and vimentin) are present in these complexes. Besides the high sensitivity of immunodetection, Western blot makes it also possible to visualize different isoforms of a certain protein.

Since not only the identification of the antigens, but also their citrullination status was of interest, the choice of antibodies for immunodetection was based on previous reports on citrullinated proteins either in serum, SF or synovial tissue of RA patients. The comparison between citrullinated proteins in serum and SF was already reported by Takizawa *et al. *[19]. In their study, soluble antigens were studied in RA serum, RA SF and osteo-arthritis (OA) SF. They could only identify citrullinated fibrinogen in RA SF. However, two years later, also citrullinated fibronectin and citrullinated vimentin were found as soluble antigens in RA SF and synovial tissue [20-22]. Citrullinated fibronectin was also detected in RA SF and synovial exosomes [23,24]. Additionally, the presence of citrullinated Fibβ and Fibγ in RA synovium has been reported by Matsuo *et al. *[25]. Based on these findings, immunodetection was performed with anti-Fibβ, anti-Fibγ, anti-fibronectin and anti-vimentin antibodies on 2D-PAGE with IC, followed by anti-modified citrulline (AMC) detection.

The citrullination of the antigens perfectly fits the model for the development and chronic nature of RA proposed by van Venrooij and Pruijn. They divided the process of autoimmunity in RA into five steps: an innocent inflammation in combination with massive apoptosis or impaired clearance can lead to the elevation of cytosolic Ca^2+ ^concentrations (1) followed by the activation of peptidylarginine deiminase (PAD) and the citrullination of proteins (2). When citrullinated antigens are presented to T cells, the production of ACPAs is triggered (3). Immune complexes can be formed if the antigens react with the auto-antibodies (4). These IC stimulate inflammatory processes (5) and cause a vicious circle of inflammation resulting in joint destruction for years [26].

This study describes the isolation and characterization of antigens residing in IC of RA patients. We found that circulating IC in the serum of RA patients and healthy controls contain Fibβ and fibronectin, both in a non-citrullinated form. In IC, isolated from RA SF, on the other hand, Fibrinogenβ, Fibrinogenγ, fibronectin and vimentin were identified. More importantly, vimentin and a minor portion of Fibβ were found to be citrullinated in the isolated complexes from RA SF. However, these citrullinated antigens were only found in IC from SF of ACPA+ RA patients, while no citrullinated antigens were found in IC from SF of ACPA- RA patients or SpA patients.

## Materials and methods

### Patients and controls

Serum and synovial fluid were collected from patients fulfilling the American College of Rheumatology criteria for RA [27] and European Spondyloarthropathy Study Group criteria for SpA [28] (for patient information see Table 1). Sera from healthy donors were used as controls. Informed consent was obtained from patients and healthy controls and the study was approved by the local ethics committee. Detailed information on the identity of the samples used in each experiment is provided in Table 1. RF titers were determined with the Waaler Rose test and ACPA titers were measured with anti-CCP-EliA (Phadia, Freiburg, Germany).

**Table 1:** Rheumatoid factor and CCP values of RA patients

	Used in experiment	diagnosis	RF (U/ml)	CCP (U/ml)
RA1	results 1&2	RA	1,280	2,839
RA2	results 1&2	RA	320	265
RA3	results 1&2	RA	0	> 1,600
SF1	results 3	RA	6	0
SF2	results 1&2&3	RA	80	1
SF3	results 3	RA	0	1
SF4	results 3	RA	0	1,6
SF5	results 3	RA	0	2
SF6	results 3	RA	5	2
SF7	results 3	RA	0	3
SF8	results 3	RA	40	4
SF9	results 3	RA	0	5
SF10	results 3	RA	0	5
SF11	results 3	RA	80	7
SF12	results 3	RA	160	10
SF13	results 1&2&3	RA	351	107
SF14	results 3	RA	183	340
SF15	results 1&2&3	RA	1,280	533
SF16	results 3	RA	1,280	608
SF17	results 3	RA	1,280	710
SF18	results 3	RA	2,560	740
SF19	results 3	RA	10,240	767
SF20	results 1	RA	1,280	1,141
SF21	results 3	RA	640	1,294
SF22	results 3	RA	0	> 1,600
SF23	results 3	RA	160	> 1,600
SF24	results 3	RA	0	> 1,600
SF25	results 3	RA	640	1,775
SF26	results 2	RA	227	ND
SF27	results 3	SpA	0	0
SF28	results 3	SpA	ND	1,6
SF29	results 3	SpA	0	2
SF30	results 3	SpA	0	2
SF31	results 3	SpA	0	3
SF32	results 3	SpA	0	4
SF33	results 3	SpA	0	11
SF34	results 3	SpA	0	ND
SF35	results 3	SpA	ND	ND
SF36	results 3	SpA	0	ND
SF37	results 3	SpA	0	ND
SF38	results 3	SpA	0	ND

The rheumatoid factor was determined with Waaler Rose (U/ml) and CCP (U/ml) values were determined with anti-CCP-EliA. Serum (RA1-RA3) as well as SF was used. The section of the article in which the sera and SF are used is mentioned in the second column. ND, not determined

### Immunoprecipitation with Immobilized Protein G

IC were further purified by affinity immunoprecipitation with Immobilized Protein G (Pierce, Rockford, IL, USA). 400 μL beads were washed twice with 500 μL phosphate buffered saline (PBS). A total of 50 μL serum or SF was mixed with 450 μL PBS and added to the beads. Sample and beads were placed on a rocker for four hours at 4°C. The beads with the bound IC were washed five times in 500 μL PBS. The pellet of protein G beads was resuspended in reducing Laemmli sample buffer for five minutes at 95°C. After centrifugation (5 minutes, 460 g) the supernatant was stored at -20°C.

Protein concentrations were determined by Coomassie (Bradford) Protein Assay (Pierce, Rockford, IL, USA) and 2 D Quant (GE Healthcare, Uppsala, Sweden).

### One-dimensional gel electrophoresis (1D-PAGE)

Protein samples were dissolved in Laemmli buffer (50 mM TrisHCl, pH 6.8, 2% SDS, 10% glycerol, bromophenol blue) with 5% β-mercapto-ethanol and incubated at 95°C for five minutes. The samples were loaded on a 10% TrisHCl polyacrylamidegel (Biorad, Hercules, CA, USA) and electrophoresis was performed by applying 150 V for 30 minutes, followed by 200 V for one hour.

### Two-dimensional gel electrophoresis (2D-PAGE)

For 2D-PAGE, protein samples were first precipitated overnight in acetone at -20°C. After centrifugation at 18,000 g for 10 minutes the samples were air dried. A total of 100 μg was resuspended in 200 μL rehydration buffer (7 M Urea, 2 M Thiourea, 2% CHAPS, 0.2% carrier ampholytes, 100 mM DTT, bromophenol blue). The sample was introduced passively in an IPG strip (11 cm, pH 4 to 7) (Biorad) as previously described [29]. Iso-electric focussing was performed in a Protean IEF Chamber (Biorad) according to the following program: 100 V, 30 minutes, linear voltage slope - 250 V, 30 minutes, linear - 500 V, one hour, linear - 1,000 V, one hour, linear - 8,000 V, four hours, rapid - 8,000 V, 35,000 V hours, rapid - 500 V, 20 h, rapid. Subsequently the strips were equilibrated in equilibration buffer (50 mM TrisHCl, pH 8.8, 6 M Urea, 20% glycerol, 2% SDS) containing 1.5% DTT for 15 minutes, followed by 4% IAA in equilibration buffer for 15 minutes.

Gel electrophoresis was carried out on a 10% TrisHCl PAGE using 150 V for 30 minutes, followed by 200 V for one hour.

### Western blot

After a 15-minute equilibration of the gels and the nitrocellulose membranes (Biorad) in CAPS (pH 11), electrophoretic transfer of proteins was performed by tank blotting in a Trans Blot Cell (Biorad), with CAPS (pH = 11), at 50 V for three hours. Successful transfer of proteins was checked by means of Ponceau S staining.

### Detection of citrullinated proteins

The presence of citrullinated proteins on the nitrocellulose blots was detected using the anti-modified citrulline (AMC) detection kit (Upstate, Charlottesville, VA, USA) according to the manufacturer's protocol. Each AMC detection was accompanied with a positive control, as indicated in the manufacturer's protocol.

### Protein identification

Visualization of proteins in the gels was performed using Sypro Ruby Protein Gel staining (Invitrogen, Carlsbad, CA, USA) for at least three hours after a 30-minute fixation in a 10% MeOH, 7% acetic acid solution. After staining, the gel was washed twice with a 10% MeOH, 7% acetic acid solution. Proteins of interest were excised from the gel and digested with modified sequence grade porcine trypsin (Promega, Madison, WI, USA) as described earlier [30]. Proteins were analyzed and identified by LC-MSMS, using a Q-TOF Ultima Mass spectrometer (Waters, Milford, MA, USA) combined with ESI source. The data were processed using Mascot Distiller and searched against the Swissprot human database, using the in-house mascot daemon searching algorithm. Identification was considered positive with a *P*-value < 0.05.

### Immunodetection

Before immunodetection, each blot was blocked for one hour in 0.3% Tween-20 in PBS. Vimentin was detected with the mouse anti-human vimentin antibody (clone V9, Sigma, St. Louis, MI, USA) at a concentration of 1/400 in 0.3% Tween-20 in PBS. After overnight incubation, vimentine was detected with HRP labelled goat anti-mouse IgG followed by ECL detection. Detection of Fibβ, Fibγ and fibronectin was performed using respectively rabbit anti-human Fibβ, rabbit anti-human Fibγ and rabbit anti-human fibronectin. Anti-rabbit HRP labelled antibody was used as a secondary antibody. ECL detection was carried out by means of Supersignal West Dura Extended Duration Substrate (Pierce).

Following each immunodetection, the blot was stripped for 30 minutes at 50°C with stripping buffer (2% SDS, 0.1 M β-mercapto-ethanol, 0.05 M Tris pH 6.8) and washed three times with 0.3% Tween20 in PBS. To check the stripping efficiency, the blot was re-incubated with secondary antibody and detected with ECL. Afterwards the blot was stripped for another 15 minutes, before incubation with a new primary antibody. Additionally, the sequence of antibodies used for immunodetection varied throughout the different experiments in order to exclude false positive results. Protein patterns were scanned and digitized using the VersaDoc Imaging System (Biorad).

## Results

### Broad range proteome approach to identify **potential antigens in RA serum and RA SF**

**Figure 1:**
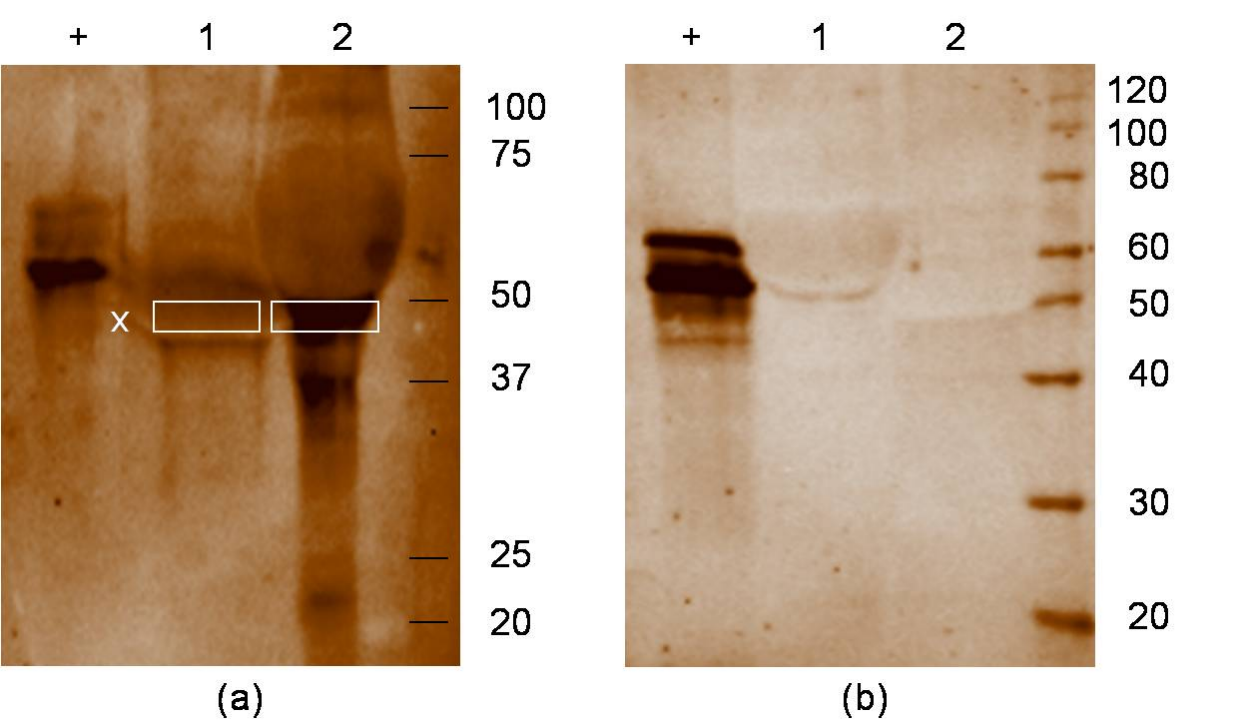
**Detection of citrullinated proteins in IC of RA SF (a) and RA serum (b) after immunoprecipitation**. 1D-PAGE and AMC staining were performed on the isolated IC and the flow through after IP, from synovial fluid and serum of RA patients. Where (+) is the positive control for AMC staining; the flow-through is shown in lane 1 and the IC-fraction in lane 2. Citrullinated proteins could be detected in the IC isolated by IP in the SF of RA patients **(a) **and were absent in the sera of RA patients **(b)**. In the fraction indicated by "x";, immunoglobulins, serum albumin and Fibβ were identified by mass spectrometry.

Immunoprecipitation (IP) was used in order to isolate the IC from serum and synovial fluid. Because of the high viscosity of SF, a hyaluronidase treatment was necessary. Both, the flow-through and the eluted IC fraction from a pool of RA SF (SF2; SF13; SF15; SF26) were subjected to 1 D gel electrophoresis (20 μg/lane). In order to identify potential autoantigens in the eluted IC fractions from SF, each lane (20 μg) from the gel was divided in 30 different plugs and analysed separately by mass spectrometry after in gel digestion. Mass spectrometric analysis revealed that the eluted IC fraction from RA SF contained mainly immunoglobulins, while almost none were detected in the flow-through fraction (data not shown). Additionally, Fibβ (at MW 50 kDa, Figure 1a box (x)) could be identified in the SF IC fraction as well as in the flow-through. At this MW, a clear positive AMC staining was detected in the isolated IC fraction from RA SF (Figure 1a lane 2), while no citrullinated proteins could be detected in the flow through of RA SF (Figure 1a lane 1). On the contrary, when the same setup was repeated with a pool of RA sera no citrullinated proteins were detected in the IC from RA sera (RA1 to RA3) (Figure 1b lane 2), while the positive control for AMC staining was explicit. In order to confirm these findings, a set of immunoblotting experiments was performed.

### Immunodetection of potential IC antigens in RA serum and SF

**Figure 2:**
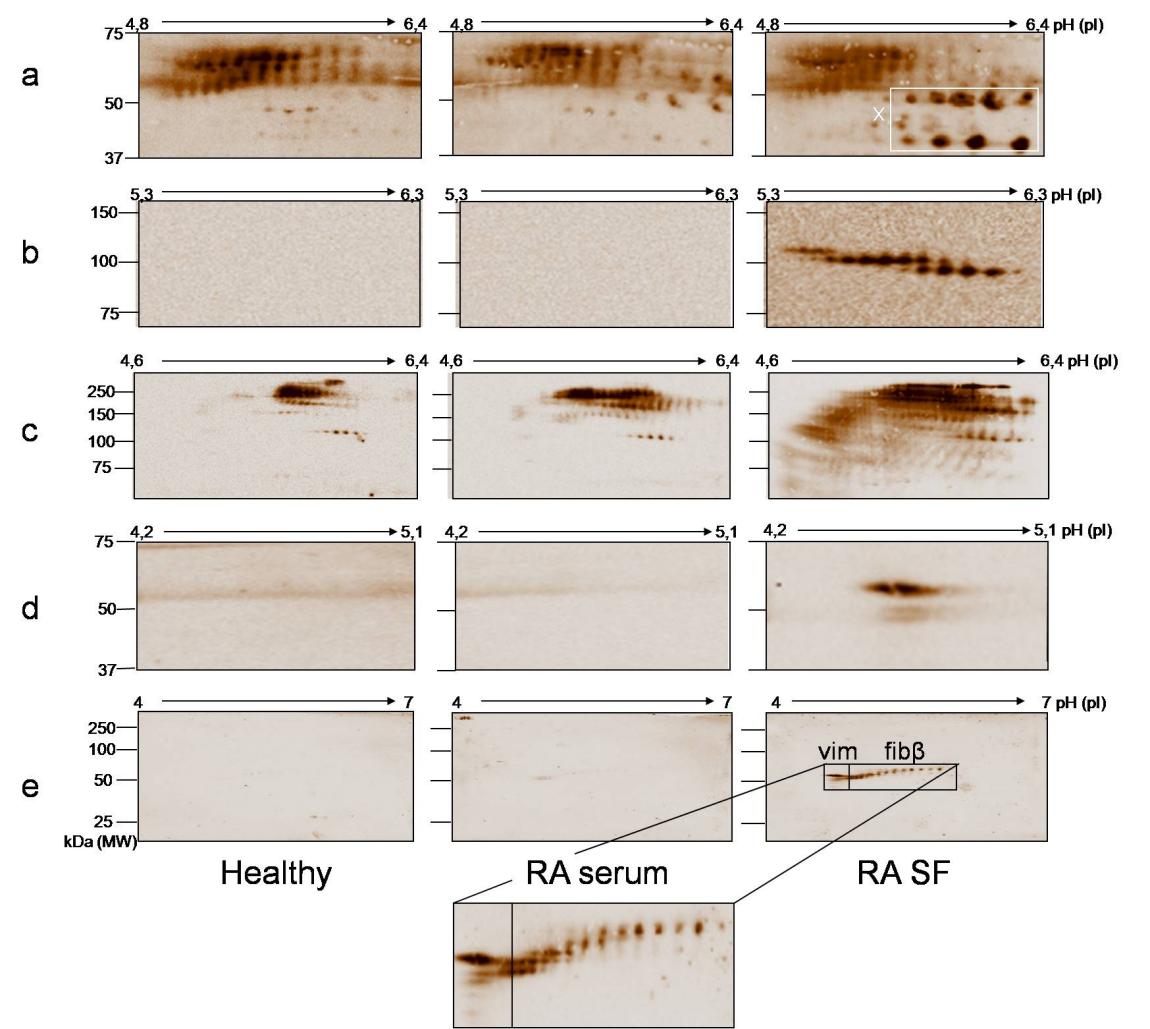
**Identification of antigens in IC of healthy serum, RA serum and RA SF by immunodetection**. 100 μg IC purified from healthy serum, RA serum and RA SF were analysed on Western blot (pH 4 to 7) and immunodetection was performed with anti-fibrinogenβ **(a)**, anti-fibrinogenγ **(b)**, anti-fibronectin **(c)**, anti-vimentin **(d) **and AMC staining **(e)**. Note that different mass and pI scales were used for clarity. Box × indicates processed isoforms of Fibβ.

First, a pool of serum obtained from healthy persons (n = 4), a pool of serum from RA patients (RA1 to RA3) and a pool of SF from RA patients (SF2; SF13; SF14; SF15) were used to isolate IC by IP. Subsequent identification of potential antigens in the isolated IC was performed by sequential immunodetection with anti-vimentin, anti-Fibβ, anti-Fibγ and anti-fibronectin on a 2D-Western blot. Between the different immunodetections, the blot was carefully stripped and adequate stripping was checked each time before subsequent primary antibody addition. The results of these experiments are summarized in Figure 2.

Fibβ was detected in IC from healthy serum and RA serum and from RA SF at a molecular weight of 50-60 kDa and pI 5-6. However in IC from RA SF, and not in IC from RA or healthy serum, some extra spots that reacted with anti-Fibβ could be detected at MW 37-50 kDa and pI 6-7. These are probably processed isoforms of Fibβ (Figure 2a, box x), which are specifically found in IC from SF.

Fibγ was visualized as three spot trains at a molecular weight around 100 kDa. As Fibγ has a molecular weight of 56 kDa, the presence of spots at 100 kDa indicated the dimeric form of Fibγ. These dimers of Fibγ were only seen in IC from RA SF and not in IC from RA serum or healthy serum (Figure 2b).

Fibronectin could be detected in IC from healthy serum, RA serum and RA SF at a molecular weight of 150-250 kDa. However, fibronectin in IC from RA SF covered a wider range of isoforms in comparison with IC from RA serum and healthy serum. It is known that fibronectin is present in biological samples in many isoforms [31]. This explains the large spreading of fibronectin protein spots in molecular weight (150-250 kDa) and pI (pH 5-7). Many more isoforms of fibronectin were observed in IC from RA SF, in comparison to IC from RA serum and healthy RA (Figure 2c). Since these extra isoforms in IC from RA SF are located at a lower molecular weight range, we presume that these isoforms are cleavage products of fibronectin.

Vimentin was detected in IC from RA SF at MW 50-60 kDa and pI 4.6 (Figure 2d). On the contrary, in healthy serum and RA serum, no vimentin was detected in the IC pool (Figure 2d).

In order to reveal citrullinated proteins, AMC detection was performed after successful stripping. In IC from healthy serum and RA serum no citrullination was observed, confirming our prior 1 D analysis. In IC from RA SF on the contrary, spots corresponding to citrullinated proteins could be detected around 50 kDa and pI 4.5-6 (Figure 2e).

To make sure that the detection of these spots was due to binding of the primary antibody and not to aspecific binding, 2D-Western blotting and immunodetection were performed using only the secondary antibodies. The few spots detected on these blots did not correspond to the spots detected with anti-vimentin, anti-Fibβ, anti-Fibγ, or anti-fibronectin (data not shown).

In order to compare the AMC results with the immunodetection results, landmarks were positioned on the blot. Using these landmarks, accurate comparison between the different stainings was possible. This comparison revealed that vimentin and Fibβ were the citrullinated proteins in IC from RA SF. Remarkably, only a minor fraction of the detected Fibβ was also positive with AMC staining. In IC from RA serum no citrullinated Fibβ or citrullinated vimentin could be detected (Figure 2e).

The observed results were confirmed in a second pool of IC from RA SF. A different sequence of antibody staining was used to minimize technical variation and duplicate blots were run to make sure that the AMC detection was not influenced by previous detections or multiple stripping steps. Again, citrullinated vimentin and some citrullinated Fibβ isoforms were present in IC from RA SF (data not shown).

### Identification of citrullinated antigens in SF of RA (CCP+ versus CCP-) and SpA

**Figure 3:**
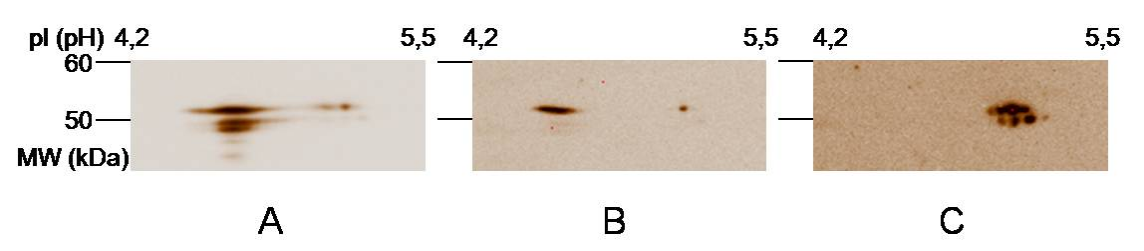
**Different isoforms of vimentin on Western blot**. Immunodetection with anti-vimentin on Western blot. **A**: shows the combination of the acidic (pI = 4.6) and the basic (pI = 5.3) isoform of vimentin; **B**: acidic isoform of vimentin; **C**: the basic isoform of vimentin. The acidic isoforms (A and B) correspond with citrullinated vimentin as detected with AMC.

Immunoprecipitation and subsequent immunodetection and AMC staining were performed on individual SF samples of 24 RA patients (12 CCP- patients: SF1 to SF12 and 12 CCP+ patients: SF13 to SF25) and 12 SpA patients (SF27 to SF38). In all the SF samples (n = 36) from RA CCP+ patients as well as RA CCP- patients and SpA patients, Fibβ and/or the processed isoforms (Figure 2a box x) could be detected. Fibγ was found in the isolated IC from 9 out of 12 CCP+ patients, 10 out of 12 CCP- patients and 9 out of 12 SpA patients (Table 2). Interestingly, vimentin was detected in half of the CCP- patients (6 out of 12) and SpA patients (7 out of 12), while 11 out of 12 CCP+ patients were positive for vimentin. The 12^th ^CCP+ patient showed a weak signal for vimentin. Vimentin could be detected in two sets of spot clusters; between 50 and 60 kDa, at pI 5.3 and pI 4.6 (Figure 3). Strikingly, the acidic isoform (pI 4.6) could only be detected in 6 out of 12 CCP+ RA patients, while none of the CCP- RA patients or SpA patients possessed this acidic isoform of vimentin. AMC staining revealed that the detected vimentin at pI 4.6 was citrullinated in five of the six patients, who were positive for the acidic isoform of vimentin. In contrast, the vimentin at pI 5.3 was not citrullinated. No citrullinated vimentin could be detected in RA CCP- and SpA patients while 5 of the 12 RA CCP+ patients contained citrullinated vimentin in the IC from SF. Citrullinated Fibβ on the other hand could only be observed in SF from one RA CCP+ patient, while no citrullinated Fibβ was detected in RA CCP- patients and SpA patients. Additionally, this citrullinated Fibβ covered only a minor portion of the detected Fibβ in IC.

**Table 2:** Immunodetection of selected antigens in IC obtained from SF of individual RA CCP+ and CCP- patients

	Patient n° (RF;CCP)	Fibβ	Fibγ	Vimentin
RA CCP-	SF1 (6;0)	+	+	+
	SF2 (80;1)	+	+	-
	SF3 (0;1)	+	+	-
	SF4 (0;1,6)	+	+	+
	SF5 (0;2)	+	+	+
	SF6 (5;2)	+	+	+
	SF7 (0;3)	+	+	+
	SF8 (40;4)	+	-	-
	SF9 (0;5)	+	+	-
	SF10 (0;5)	+	-	-
	SF11 (80;7)	+	+	+
	SF12 (160;10)	+	+	-
				
RA CCP+	SF13 (351;107)	+*	-	+
	SF14 (183;340)	+	-	+
	SF15 (1,280;533)	+	-	+*
	SF16 (1,280;608)	+	+	+*
	SF17 (1,280;710)	+	+	+
	SF18 (2,560;740)	+	+	+
	SF19 (10,240;767)	+	+	weak
	SF21 (640;1294)	+	+	+
	SF22 (0;1,600)	+	+	+*
	SF23 (160;1,600)	+	+	+*
	SF24 (0;1,600)	+	+	+
	SF25 (640;1,775)	+	+	+*
				
SpA	SF27 (0;0)	+	-	-
	SF28 (ND;1,6)	+	+	+
	SF29 (0;2)	+	+	+
	SF30 (0;2)	+	+	+
	SF31 (0;3)	+	+	+
	SF32 (0;4)	+	+	-
	SF33 (0;11)	+	+	-
	SF34 (0;ND)	+	+	-
	SF35 (ND;ND)	+	+	-
	SF36 (0;ND)	+	-	+
	SF37 (0;ND)	+	-	+
	SF38 (0;ND)	+	+	+

(+) Indicates the positive immunodetection; (-) indicates absence of the positive antibody staining and (*) indicates that the antigen was citrullinated as confirmed by AMC staining. ND, not determined

These results indicate that citrullinated antigens (mainly vimentin) present in IC of SF from RA patients can only be found in CCP+ patients and not in CCP- patients or SpA patients.

## Discussion

One of the purpose of this study was to identify the antigens in IC from serum of RA patients. However, the joint is the primary target in the pathology of RA and high amounts of antibodies [32,33], citrullinated proteins [19-21], and IC [10,34] are found in RA synovial fluid. Moreover, Wipke *et al*. showed that IC in the joint are necessary to initiate inflammation [8]. Investigation of the IC in SF could thus be even more informative than analysing serum or plasma.

In order to isolate IC, immunoprecipitation was performed on serum and SF. However, a major drawback of this technique is the co-purification of high amounts of free immunoglobulins. Previous experiments had already shown that this contaminating factor caused interference during further identification of the antigen by mass spectrometry (data not shown). To separate immunoglobulins from other proteins, 1D-PAGE in combination with in gel digestion and mass spectrometry was performed as a broad range proteome approach in order to detect new potential antigen in IC. Despite the high abundance of immunoglobulins in the purified IC, Fibβ was detected in the IC of RA SF as well as in the flow-through. At the MW of Fibβ, a clear positive AMC staining on Western blot was detected in the isolated IC fraction, while no citrullinated proteins were detected in the flow-through. This indicates that Fibβ could be present in a citrullinated form in IC in SF of RA patients. In RA serum, however, no citrullinated proteins were detected in the IC fraction. This was in contrast to the findings of Zhao *et al. *[15] . They reported the presence of citrullinated Fibβ in IC from plasma of CCP+ RA patients and confirmed their presence in synovial tissue. The discrepancy in results might be explained by the difference in isolation method and the fact that they worked with plasma instead of serum. Indeed because serum does not contain any clotting factors, some of the fibrinogen, citrullinated or native, free or in immune complexes, might have already been removed during clotting. Additionally, patient variability or disease status variation can not be excluded as contributing factors.

By means of this broad range approach, Fibβ, possibly citrullinated, was found as an antigen in SF from RA patients. However, it should be noted that due to the low abundance of the antigens and the excess of immunoglobulins, some antigens will not be detected in this approach, especially the antigens that co-migrate with immunoglobulins on 1 D SDS PAGE.

Therefore, in a second approach, 2 D PAGE was combined with immunodetection to overcome the interference of the immunoglobulins as well as the low abundance of the antigens. By means of 2 D PAGE and immunodetection we wanted to confirm the previous results from the broad proteome approach and also analyse the presence of well-known antigens in RA, such as Fibβ, Fibγ, fibronectin and vimentin.

Fibβ and fibronectin were found in IC obtained from healthy serum, RA serum and RA SF, indicating that the presence of Fibβ and fibronectin in IC is not specific for RA or RA SF. Fibγ and vimentin could only be detected in the IC from RA SF (Figure 2). The extra isoforms of Fibβ (Figure 2a, box x) were also exclusively found in SF. Besides the presence/identification of the antigens in IC, the citrullination status of these antigens was analyzed. No citrullinated proteins were detected in IC of RA serum, while RA SF contained different citrullinated proteins, which confirmed previous 1D-PAGE analysis. The citrullinated proteins in IC from RA SF were identified as vimentin and Fibβ. The fact that vimentin was found as antigen during 2 D analysis and not during the broad range proteome approach with 1 D SDS PAGE, can be explained by the fact that vimentin has the same molecular weight as the heavy chain of immunoglobulins. The detection of citrullinated Fibβ on 2D-PAGE confirmed our results from the broad range approach. During later analysis we found that citrullinated Fibβ could only be detected in patient 13 (SF13) (Table 2), which was present in the pool for 1D-PAGE as well as the pool for 2D-PAGE. However, it should be noted that fibrinogen, citrullinated or not, could possibly be deposited in synovium tissue and thereby not, or in a lesser extend, be detected in synovial fluid.

Next, we analysed individual SF samples from CCP+ RA patients and CCP- RA patients. SpA patients were included as disease controls. Since fibronectin was present in healthy serum as well as RA serum and RA SF, we focussed on Fibβ, Fibγ and vimentin during further analysis. When individual samples of CCP+, CCP- and SpA synovial fluid were processed, we observed absence of citrullinated proteins in IC in the CCP- patients and SpA patients (n = 24) while half of the CCP+ patients contained citrullinated vimentin or Fibβ.

Citrullinated fibrinogen is known to be present in SF of RA patients [25]. Our data showed that in IC of SF only Fibβ was citrullinated and that Fibγ was present exclusively in IC of SF, but in a non-citrullinated form. The Fibβ isoforms detected on the AMC blot of one patient, however, were only a minor fraction of the Fibβ isoforms detected with anti-Fibβ. The lower abundance of Fibβ could be due to residing fibrinogen in the synovial tissue.

A remarkable difference between our data and previous reports is the presence of citrullinated vimentin in IC of CCP+ RA SF. This antigen could not be detected in IC of healthy or RA serum. Citrullinated vimentin is known to be an important antigen in RA [35] and is present in synovial fluid [21], but its presence in IC has not been reported. Moreover, no citrullinated antigens were found in IC of SF from our control groups, consisting of CCP- RA patients and SpA patients.

Since CCP+ patients have a more destructive course of disease and because CCP+ patients contain citrullinated vimentin in their IC, we hypothesize that citrullinated vimentin plays an important pathophysiological role in the perpetuation of RA. Moreover, in contrast to Fibβ, vimentin is an intracellular antigen and therefore not expected in IC. Additionally, the presence of intracellular citrullinated proteins in the synovium is specific for RA, while extracellular citrullinated proteins lack this specificity [36]. These RA specific synovial intracellular citrullinated proteins are also associated with significantly higher systemic and local ACPA levels and with local ACPA production in the joint [36].

## Conclusions

Our data reveal the presence of citrullinated vimentin and a less pronounced presence of citrullinated Fibβ in RA SF (of CCP+ patients), while no citrullinated proteins could be detected in IC from RA serum and healthy serum or in IC from SF of RA CCP- patients and SpA patients. Combining these findings with the five-point circle of van Venrooij [26] we conclude that CCP+ RA patients are more susceptible to the perpetuation of inflammation and possibly have a more severe disease state because of the presence of citrullinated vimentin and Fibβ in their SF IC. Taken together, our data indicate that citrullinated vimentin is an important antigen in IC of CCP+ RA patients and therefore implies its importance in the pathology of RA.

## Abbreviations

2D-PAGE: two dimensional polyacrylamide gelelectrophoresis; ACPA: anti-citrullinated protein/peptide antibody; AMC: anti-modified citrulline; CAPS: 3-(cyclohexylamino)-1-propanesulfonic acid; CCP: anti-cyclic citrullinated peptide; CHAPS: 3-((3-Cholamidopropyl)dimethylammonio)-1-propanesulfonate; DTT: dithiothreitol; ECL: enhanced chemiluminescence; ELISA: enzyme linked immunosorbent assay; ESI: electrospray ionisation; Fib: fibrinogen; GM-CSF: granulocyte-macrophage colony-stimulating factor; HRP: horse radish peroxidise; IAA: iodoacetamide; IC: immune complex; Ig: immunoglobulin; IL: interleukin; IP: immunoprecipitation; IPG: immobilized pH gradient; LC: liquid chromatography; M: molar; MS: mass spectrometry; MW: molecular weight; NO: nitrogen oxide; OA: osteo-arthritis; PAD: peptidyl arginine deiminase; PBMC: peripheral blood mononuclear cells; PBS: phosphate buffer saline; PEG: polyethylene glycol; pI: iso-electric point; Q-TOF: quadrupole time of flight; RA: rheumatoid arthritis; RF: rheumatoid factor; SDS: sodium dodecyl sulphate; SDS-PAGE: sodium dodecyl sulphate-polyacrylamide gelelectrophoresis; SF: synovial fluid; SpA: Spondyloarthropathy; TNFα: tumor necrosis factor.

## Competing interests

The authors declare that they have no competing interests.

## Authors' contributions

KVS performed most of the practical work and data-analysis and wrote the manuscript. KT assisted in performing ultracentrifugation, immunoprecipitation and Western blot analysis. She also helped to draft the manuscript. MDC gave practical assistance during the experiments. DE, FDK and DD helped in the design of the study and the critical analysis of the data. All authors read and approved the manuscript.

## Authors' information

The locations where the authors' completed their education follow: KVS, PharmD, Ghent University, Belgium; KT, MSc, PhD, Ghent University Hospital, Belgium; MDC, PharmD, Ghent University, Belgium; DE, MD, PhD, Ghent University Hospital, Belgium; FDK, MD, PhD, Ghent University Hospital, Belgium; DD, PharmD, PhD, Ghent University, Belgium

## References

[B1] Alamanos Y Drosos AA Epidemiology of adult rheumatoid arthritis Autoimmun Rev 2005 4 130 136 10.1016/j.autrev.2004.09.002 15823498

[B2] Klareskog L Ronnelid J Lundberg K Padyukov L Alfredsson L Immunity to citrullinated proteins in rheumatoid arthritis Annu Rev Immunol 2008 26 651 675 10.1146/annurev.immunol.26.021607.090244 18173373

[B3] Weissmann G The pathogenesis of rheumatoid arthritis Bull NYU Hosp Jt Dis 2006 64 12 15 17121483

[B4] Weissmann G Pathogenesis of rheumatoid arthritis J Clin Rheumatol 2004 10 S26 31 10.1097/01.rhu.0000130687.75646.44 17043497

[B5] Dorner T Egerer K Feist E Burmester G Rheumatoid factor revisited Current Opinion In Rheumatology 2004 16 246 253 10.1097/00002281-200405000-00013 15103252

[B6] Scrivo R Di Franco M Spadaro A Valesini G The immunology of rheumatoid arthritis Ann N Y Acad Sci 2007 1108 312 322 10.1196/annals.1422.033 17893995

[B7] Shmagel KV Chereshnev VA Molecular bases of immune complex pathology Biochemistry (Mosc) 2009 74 469 479 10.1134/S0006297909050010 19538120

[B8] Wipke BT Wang Z Nagengast W Reichert DE Allen PM Staging the initiation of autoantibody-induced arthritis: a critical role for immune complexes J Immunol 2004 172 7694 7702 15187152 10.4049/jimmunol.172.12.7694

[B9] Jarvis JN Wang W Moore HT Zhao L Xu C *In vitro *induction of proinflammatory cytokine secretion by juvenile rheumatoid arthritis synovial fluid immune complexes Arthritis Rheum 1997 40 2039 2046 10.1002/art.1780401117 9365094

[B10] Schuerwegh AJ Dombrecht EJ Stevens WJ Van Offel JF Kockx MM Bridts CH De Clerck LS Synovial fluid and peripheral blood immune complexes of patients with rheumatoid arthritis induce apoptosis in cytokine-activated chondrocytes Rheumatol Int 2007 27 901 909 10.1007/s00296-007-0336-3 17404735

[B11] Mathsson L Lampa J Mullazehi M Ronnelid J Immune complexes from rheumatoid arthritis synovial fluid induce FcgammaRIIa dependent and rheumatoid factor correlated production of tumour necrosis factor-alpha by peripheral blood mononuclear cells Arthritis Res & Ther 2006 8 R64 16569263 10.1186/ar1926 PMC1526644

[B12] Gioud-Paquet M Auvinet M Raffin T Girard P Bouvier M Lejeune E Monier JC IgM rheumatoid factor (RF), IgA RF, IgE RF, and IgG RF detected by ELISA in rheumatoid arthritis Ann Rheum Dis 1987 46 65 71 10.1136/ard.46.1.65 3813676 PMC1002060

[B13] De Rycke L Peene I Hoffman IE Kruithof E Union A Meheus L Lebeer K Wyns B Vincent C Mielants H Boullart L Serre G Veys EM De Keyser F Rheumatoid factor and anticitrullinated protein antibodies in rheumatoid arthritis: diagnostic value, associations with radiological progression rate, and extra-articular manifestations Ann Rheum Dis 2004 63 1587 1593 10.1136/ard.2003.017574 15547083 PMC1754859

[B14] Renaudineau Y Jamin C Saraux A Youinou P Rheumatoid factor on a daily basis Autoimmunity 2005 38 11 16 10.1080/08916930400022574 15804700

[B15] Zhao X Okeke NL Sharpe O Batliwalla FM Lee AT Ho PP Tomooka BH Gregersen PK Robinson WH Circulating immune complexes contain citrullinated fibrinogen in rheumatoid arthritis Arthritis Res Ther 2008 10 R94 10.1186/ar2478 18710572 PMC2575608

[B16] Schellekens GA Visser H de Jong BA van den Hoogen FH Hazes JM Breedveld FC van Venrooij WJ The diagnostic properties of rheumatoid arthritis antibodies recognizing a cyclic citrullinated peptide Arthritis Rheum 2000 43 155 163 10.1002/1529-0131(200001)43:1<155::AID-ANR20>3.0.CO;2-3 10643712

[B17] Robinson MW Scott DG Bacon PA Walton KW Coppock JS Scott DL What proteins are present in polyethylene glycol precipitates from rheumatic sera? Ann Rheum Dis 1989 48 496 501 10.1136/ard.48.6.496 2742403 PMC1003794

[B18] McDougal JS Redecha PB Inman RD Christian CL Binding of immunoglobulin G aggregates and immune complexes in human sera to Staphylococci containing protein A J Clin Invest 1979 63 627 636 10.1172/JCI109345 438327 PMC371997

[B19] Takizawa Y Suzuki A Sawada T Ohsaka M Inoue T Yamada R Yamamoto K Citrullinated fibrinogen detected as a soluble citrullinated autoantigen in rheumatoid arthritis synovial fluids Ann Rheum Dis 2006 65 1013 1020 10.1136/ard.2005.044743 16449316 PMC1798256

[B20] Tabushi Y Nakanishi T Takeuchi T Nakajima M Ueda K Kotani T Makino S Shimizu A Hanafusa T Takubo T Detection of citrullinated proteins in synovial fluids derived from patients with rheumatoid arthritis by proteomics-based analysis Ann Clin Biochem 2008 45 413 417 10.1258/acb.2007.007205 18583628

[B21] Tilleman K Van Steendam K Cantaert T De Keyser F Elewaut D Deforce D Synovial detection and autoantibody reactivity of processed citrullinated isoforms of vimentin in inflammatory arthritides Rheumatology (Oxford) 2008 47 597 604 10.1093/rheumatology/ken077 18326534

[B22] Chang X Yamada R Suzuki A Kochi Y Sawada T Yamamoto K Citrullination of fibronectin in rheumatoid arthritis synovial tissue Rheumatology (Oxford) 2005 44 1374 1382 10.1093/rheumatology/kei023 16105911

[B23] Sawada T Hashimoto S Kanzaki T Suzuki A Yamada R Shoji A Hayashi H Tahara K Yamamoto K Identification of citrullinated antigen as component of immune complex in synovial fluids from patients with rheumatoid arthritis Arthritis and Rheumatism 2008 58 S520 S520

[B24] Skriner K Adolph K Jungblut PR Burmester GR Association of citrullinated proteins with synovial exosomes Arthritis Rheum 2006 54 3809 3814 10.1002/art.22276 17133577

[B25] Matsuo K Xiang Y Nakamura H Masuko K Yudoh K Noyori K Nishioka K Saito T Kato T Identification of novel citrullinated autoantigens of synovium in rheumatoid arthritis using a proteomic approach Arthritis Res Ther 2006 8 R175 10.1186/ar2085 17125526 PMC1794520

[B26] van Venrooij WJ Pruijn GJ An important step towards completing the rheumatoid arthritis cycle Arthritis Res Ther 2008 10 117 10.1186/ar2504 18828887 PMC2592789

[B27] Arnett F Edworthy S Bloch D Mcshane D Fries J Cooper N Healey L Kaplan S Liang M Luthra H Medsger T Mitchell D Neustadt D Pinals R Schaller J Sharp J Wilder R Hunder G The American-Rheumatism-Association 1987 Revised Criteria For The Classification Of Rheumatoid-Arthritis Arthritis and Rheumatism 1988 31 315 324 10.1002/art.1780310302 3358796

[B28] Dougados M van der Linden S Juhlin R Huitfeldt B Amor B Calin A Cats A Dijkmans B Olivieri I Pasero G The European Spondylarthropathy Study Group preliminary criteria for the classification of spondylarthropathy Arthritis Rheum 1991 34 1218 1227 10.1002/art.1780341003 1930310

[B29] Rabilloud T Valette C Lawrence JJ Sample application by in-gel rehydration improves the resolution of two-dimensional electrophoresis with immobilized pH gradients in the first dimension Electrophoresis 1994 15 1552 1558 10.1002/elps.11501501223 7536671

[B30] Tilleman K Van Beneden K Dhondt A Hoffman I De Keyser F Veys E Elewaut D Deforce D Chronically inflamed synovium from spondyloarthropathy and rheumatoid arthritis investigated by protein expression profiling followed by tandem mass spectrometry Proteomics 2005 5 2247 2257 10.1002/pmic.200401109 15846842

[B31] Przybysz M Borysewicz K Szechinski J Katnik-Prastowska I Synovial fibronectin fragmentation and domain expressions in relation to rheumatoid arthritis progression Rheumatology (Oxford) 2007 46 1071 1075 10.1093/rheumatology/kem067 17459959

[B32] Rodriguez-Bayona B Perez-Venegas JJ Rodriguez C Brieva JA CD95-Mediated control of anti-citrullinated protein/peptides antibodies (ACPA)-producing plasma cells occurring in rheumatoid arthritis inflamed joints Rheumatology (Oxford) 2007 46 612 616 10.1093/rheumatology/kel395 17132692

[B33] Snir O Widhe M Hermansson M von Spee C Lindberg J Hensen S Lundberg K Engstrom A Venables PJ Toes RE Holmdahl R Klareskog L Malmstrom V Antibodies to several citrullinated antigens are enriched in the joints of rheumatoid arthritis patients Arthritis Rheum 2010 62 44 52 10.1002/art.25036 20039432

[B34] Agarwal V Misra R Aggarwal A Immune complexes contain immunoglobulin A rheumatoid factor in serum and synovial fluid of patients with polyarticular juvenile rheumatoid arthritis Rheumatology (Oxford) 2002 41 466 467 10.1093/rheumatology/41.4.466 11961181

[B35] Vossenaar ER Despres N Lapointe E van der Heijden A Lora M Senshu T van Venrooij WJ Menard HA Rheumatoid arthritis specific anti-Sa antibodies target citrullinated vimentin Arthritis Res Ther 2004 6 R142 150 10.1186/ar1149 15059278 PMC400433

[B36] De Rycke L Nicholas AP Cantaert T Kruithof E Echols JD Vandekerckhove B Veys EM De Keyser F Baeten D Synovial intracellular citrullinated proteins colocalizing with peptidyl arginine deiminase as pathophysiologically relevant antigenic determinants of rheumatoid arthritis-specific humoral autoimmunity Arthritis Rheum 2005 52 2323 2330 10.1002/art.21220 16052592

